# Multi-faceted regulation of CREB family transcription factors

**DOI:** 10.3389/fnmol.2024.1408949

**Published:** 2024-08-06

**Authors:** Md Arifur Rahman Chowdhury, Md Mazedul Haq, Jeong Hwan Lee, Sangyun Jeong

**Affiliations:** ^1^Department of Bioactive Material Sciences, Jeonbuk National University, Jeonju, Republic of Korea; ^2^Department of Molecular Biology, and Research Center of Bioactive Materials, Jeonbuk National University, Jeonju, Republic of Korea; ^3^Division of Life Sciences, Jeonbuk National University, Jeonju, Republic of Korea

**Keywords:** cAMP responsive element, CREB, alternative splicing, transcriptional co-activator, post-transcriptional modification, post-translational modification, epigenetic modification

## Abstract

cAMP response element-binding protein (CREB) is a ubiquitously expressed nuclear transcription factor, which can be constitutively activated regardless of external stimuli or be inducibly activated by external factors such as stressors, hormones, neurotransmitters, and growth factors. However, CREB controls diverse biological processes including cell growth, differentiation, proliferation, survival, apoptosis in a cell-type-specific manner. The diverse functions of CREB appear to be due to CREB-mediated differential gene expression that depends on cAMP response elements and multi-faceted regulation of CREB activity. Indeed, the transcriptional activity of CREB is controlled at several levels including alternative splicing, post-translational modification, dimerization, specific transcriptional co-activators, non-coding small RNAs, and epigenetic regulation. In this review, we present versatile regulatory modes of CREB family transcription factors and discuss their functional consequences.

## 1 Introduction

cAMP response element-binding protein (CREB) is a nuclear transcription factor that contain a conserved basic region/leucine zipper (bZIP) domain and involved in a wide range of biological processes, precisely in learning, memory, stress response and addiction (Hai and Hartman, 2001; Chowdhury et al., 2023). The protein sequences, regulatory consequences, or even cellular and biological roles of CREB repeatedly conserved over the species, which was first identified in 1987 as a cAMP-responsive transcription factor that controls the *somatostatin* gene (Montminy et al., 1986, 1990; Montminy and Bilezikjian, 1987; Yamamoto et al., 1988). The regulatory mode of CREB is a complex multi-step process, involving signal activation, signal transduction, protein kinase activation, CREB phosphorylation, dimerization, DNA binding, and transcriptional regulation. This complexity permits explicit regulation and fine-tuning of gene expression.

In response to a variety of extracellular stimuli the activated kinases, such as protein kinase A (PKA), Ca^2+^/calmodulin-dependent protein kinases (CaMKs), mitogen- and stress-activated protein kinases (MSKs), and ribosomal protein S6 kinases (RSKs), phosphorylate the multiple phosphorylation sites in the kinase-inducible domain (KID), leading to either activation or inactivation of CREB. Here, the physiological stimuli that activate or inhibit CREB include hormones (insulin), BDNF, EGF, neurotransmitters (dopamine and glutamate), intracellular second messengers (cAMP and Ca^2+^), neural activity, sensory stimuli (light and smell), and different types of stressors (exercise, toxins, and UV radiation) (Wang et al., 2018; Imoto et al., 2020; Narasimhamurthy et al., 2022; Chowdhury et al., 2023; Nayar et al., 2024). The detailed CREB-mediated signaling pathway and its functional roles have been well reviewed elsewhere (Manning et al., 2017; Chowdhury et al., 2023). The conserved basic leucine zipper domain mediates dimerization to form a homodimer or heterodimer (Gonzalez et al., 1989; Meyer et al., 1993). The active dimer of the CREB/activating transcription factor (ATF) family binds to the conserved sequences (5′-TGACGT(C/G) A-3′ or 5′-CGTCA-3′ in human and 5′-GTGAC-GT(A/C) (A/G)-3′ in *Drosophila*) in the promoter regions of target genes, named as *cis*-acting cAMP responsive elements (CREs) and subsequently CREB recruits co-activators like CREB binding protein (CBP) or p300, leading to additional recruitment of the RNA polymerase complex and the initiation of transcription (Deutsch et al., 1988; Roesler et al., 1988; Chrivia et al., 1993).

Experimental studies demonstrate that CREB is involved in cell growth through regulation of *cyclin D1* and *c-myc*, cell differentiation through *GATA-1/GATA-2* and *NRF-2*, cell survival through *Bcl-2*, *Bcl-xL*, and *MCL-1* as well as cell metabolism through glucokinase and fatty acid synthase (Wilson et al., 1996; Blobel et al., 1998; Wang et al., 1999; Katoh et al., 2001; Boulon et al., 2002; Jiang et al., 2008). Therefore, dysregulation of CREB alters expression levels of target genes, affecting many different aspects of cellular functions, development and balanced physiology.

In this review we first address the functional properties of CREB structural components and functional differences of distinguished splice isoforms. We also discuss how kinase-mediated phosphorylation or post-translational modification of CREB alters its binding affinity, dimerization property, and recruitment of co-activators, and finally we summarize how these complexes promote or suppress the transcription of target genes (Table 1) to exert biological functions.

**TABLE 1:** Potential target genes of the *Drosophila* CREB.

Functional categories	Target genes	References
Circadian rhythm	*period (per)*	Reddy et al., 1984; Jackson et al., 1986; Helfrich-Förster, 2000
	*timeless (tim)*	
	*clock (Clk)*	Citri et al., 1987; Allada et al., 1998; Darlington et al., 1998
	*cycle (cyc)*	Rutila et al., 1998
	*double-time (dbt)*	Kloss et al., 1998; Price et al., 1998
Development	*ultrabithorax (Ubx)*	Thüringer et al., 1993; Eresh et al., 1997
Lipogenesis/cholesterol regulation	*Sterol regulatory element binding protein (SREBP)*	Dooley et al., 1999
Cardiac development	*tinman (tin)*	Venkatesh et al., 2000
Drug tolerance/ behavioral tolerance	*Slowpoke (Slo)*	Wang et al., 2009
Sleep homeostasis	*Disrupted-in-Schizophrenia-1 (DISC1)*	Sawamura et al., 2008
Sleep-wake states	*homer*	Naidoo et al., 2012
Learning and memory	*activin, homer, and staufen*	Miyashita et al., 2012
	*staufen, orb, moesin, translation initiation factor 2 subunit gamma (Eif-2G), oskar and eukaryotic initiation factor 5C (Eif-5C)*	Dubnau et al., 2003
	*Fragile X messenger ribonucleoprotein 1 (fmr1)*	Kanellopoulos et al., 2012
Synaptic growth	*Fasciclin II (Fas2)*	Schuster et al., 1996
Fasting-induced genes	*long non-coding RNA: *CR45018*, Limostatin (*Lst)*, Phosphoribosylformylglycinamidine synthase (Pfas)/*ade2*, Imaginal morphogenesis protein-Late 2 *(ImpL2)*, phosphatidate phosphatase (*CG11425)*, glycine N-methyltransferase *(Gnmt)*, Imaginal disc growth factor 1 (*Idgf1)*, sarcosine dehydrogenase *(Sardh)*, adenylosuccinate lyase *(AdSL)*, glutaminase *(GLS)*, Phosphoenolpyruvate carboxykinase 2 (*Pepck2)*, NAD-dependent methylenetetrahydrofolate dehydrogenase *(Nmdmc)*, Serine racemase *(Srr)*, pugilist *(pug)*, Adenosine deaminase-related growth factor D (*Adgf-D), Thor*, Branched chain amino acid transaminase (Bcat)*	Wang T. et al., 2021

## 2 Structural components

**FIGURE 1:**
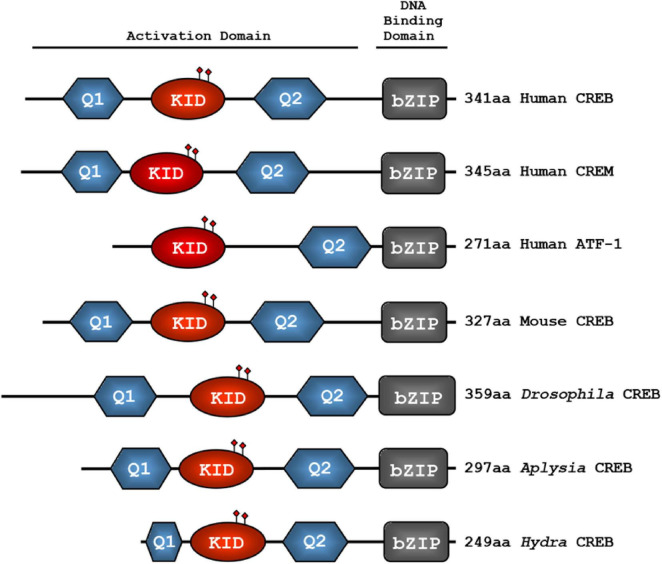
Evolutionarily conserved domains of CREB family transcription factors. The glutamine-rich domains (Q1 and Q2), the kinase-inducible domain (KID), and the basic region/leucine zipper domain (bZIP) are conserved from hydra to human. The KID domain contains several functional phosphorylation sites which are shown in closed diamond shape. The basic region mediates the binding to CRE sequences, whereas the leucine zipper domain induces dimerization. The Q1-KID-Q2 domains together provide transcriptional activation function.

Mammals have been identified with more than 20 proteins named with the CREB/ATF prefix (Cui et al., 2021), while *Drosophila melanogaster* has two CREB proteins termed dCREBA and dCREBB/CREBB-17A (Yin et al., 1995a,b; Andrew et al., 1997). The dCREBB/CREBB-17A is more homologous to mammalian CREB and cAMP responsive element modulator (CREM) proteins, whereas dCREBA is far less conserved (Eisenhardt et al., 2003; Shanware et al., 2010). These conserved CREB proteins share common structural domains, and each of those domains is capable of regulating transcription (Figure 1). The DNA-binding and dimerization domains at the C-terminus of CREB protein are named bZIP domains (Figure 1). Multiple phosphorylation sequences are known as KID domains (Bartsch et al., 1998; Luo et al., 2012), and glutamine rich 1 (Q1) and 2 (Q2) domains are considered to be constitutive active domains (CADs) of CREB proteins (Sandoval et al., 2009). Functionally, the bZIP domain facilitates binding and dimerization, while stimulus-dependent phosphorylation of the KID domain initiates co-activator interaction. Q1 and Q2 domains interact with the basal transcriptional machinery, facilitating CRE-driven expression that is independent of stimulus (Hoeffler et al., 1988; Gonzalez and Montminy, 1989; Hai et al., 1989; Foulkes et al., 1991). The alignment of multiple CREB/ATF-1 or CREM family proteins with numerous studies suggests that CREB, as an activator, has a domain organization of Q1-KID-Q2-bZIP with consistent exon usage (Figure 1; Lalli et al., 1996; Zhang D. et al., 2022).

**FIGURE 2:**
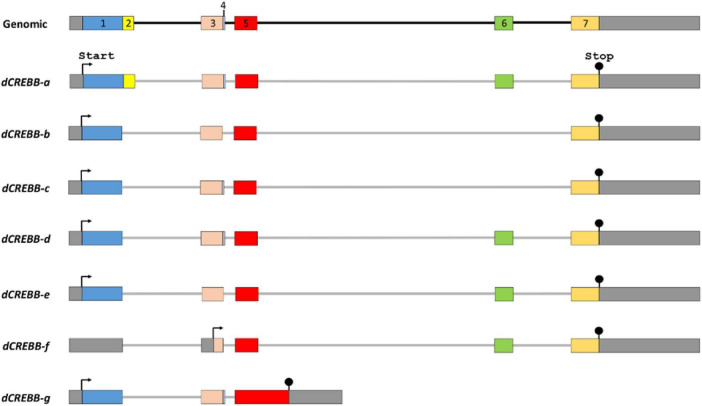
Alternative splicing of the *dCREBB* gene. Alternative splicing produces at least 7 different protein isoforms from a single *dCREBB* gene in *Drosophila*. The protein dCREBB-a contains Q1, Q2, KID, and bZIP domains, whereas the isoform dCREBB-b lacks Q2 domain. Each arrow indicates the start codon (ATG). Stop codon (TAA) is marked by “Stop.”

The repressor isoforms lack the Q (Q1 or Q2) and KID domains, for examples: inducible cAMP early repressor (ICER) and cAMP responsive element modulator (S-CREM) are the repressor isoforms of human CREB and CREM, respectively (Walker et al., 1994; Borlikova and Endo, 2009). Interestingly, in *Drosophila*, both activator and repressor isoforms share the Q1-KID-Q2-bZIP domains, except for a few exons (presence or absence of exon 2, 4, or 6) (Figure 2; Girardet et al., 1996; Poels et al., 2004). On the basis of the sequence orientation of CREB genes, four major functional domains from the N terminal to C terminal and nuclear localization signaling peptide are described below:

Glutamine rich 1 (Q1) domain−the Q1 domain is required for transactivation. Intensive studies demonstrate that Q1 has no direct role in the recruitment of co-activators or RNA polymerase II initiation complex (Mayr et al., 2001). However, collectively Q1 and Q2 domains interact with TATA binding protein-associated factor II 135 (TAFII135) and CCAAT/enhancer binding proteins (C/EBPs) to stimulate overall transcription (Horikoshi et al., 1988; Felinski and Quinn, 1999; Chen et al., 2003).

**FIGURE 3:**
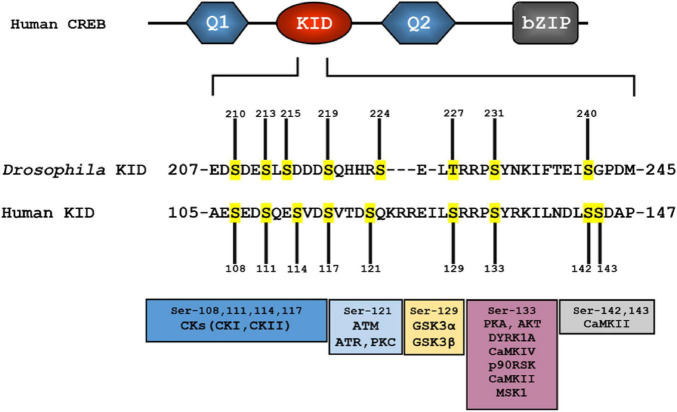
Several phosphorylation sites (in yellow) observed in the human kinase-inducible domain (KID) appear to be conserved in the *Drosophila* KID. Multiple protein kinases that can phosphorylate specific serine residue(s) are shown in the bottom. Homologous protein kinases to some of human protein kinases are also found in *Drosophila* (see Table 2).

Kinase inducible domain (KID)−contains multiple phosphorylation sites and modulates transcription upon context-dependent phosphorylation by several kinases (Figure 3; Kwok et al., 1994). Specifically, phosphorylation of Ser-133 in the KID domain of CREB increases its binding affinity for the kinase-inducible domain interacting (KIX) domain of the co-activator CREB-binding protein (CBP). The active interaction between phospho-KID and KIX triggers the transcription machinery to bind to CREs, and modulates the transcription of genes to exert biological functions, such as circadian rhythm regulation, and long-term memory (Eckner et al., 1994; Parker et al., 1996; Radhakrishnan et al., 1997; Zor et al., 2002).

Glutamine rich 2 (Q2) domain−is responsible for recognizing and binding to the canonical CRE, as well as binding to the RNA polymerase II initiation complex. This constitutive activation domain (CAD) interacts with and recruits the promoter recognition factor TFIID/TAFII130 (Ferreri et al., 1994; Altarejos and Montminy, 2011). Both KID and Q2 domains, collectively known as transactivation domain, are required for basal transcription, and trigger CRE-driven gene expression independently of external stimuli (Zor et al., 2002; Conkright et al., 2003b). Mutations in Q2 impair the overall basal transcriptional activity of CREB (Brindle et al., 1993; Ferreri et al., 1994). Collectively, the two glutamine rich domains Q1 and Q2 are essential for basal transactivation activity (Martinez-Yamout et al., 2023).

Basic/leucine zipper domain (bZIP)−is required for dimerization and binding to the consensus CRE region (Montminy et al., 1986; Delegeane et al., 1987; Hardy and Shenk, 1988; Yamamoto et al., 1988; Hurst et al., 1990; Sassone-Corsi, 1998). Site-directed mutational analysis indicates that this leucine zipper domain is critical for the formation of CREB protein homodimer and transcriptional activity (Behr and Weinbauer, 2000; Craig et al., 2001; Poels and Vanden Broeck, 2004). The binding affinity of CREB to DNA can be modulated by Mg^2+^ ions; an increase in Mg^2+^ concentration leads to a 2-fold increase in the binding of phosphorylated CREB to the CRE-binding site, whereas Mg^2+^ ions facilitate bZIP’s affinity for CRE by more than 25-fold and potentiate gene expression (Schumacher et al., 2000; Moll et al., 2002). Interstingly a recent study demotsrate that CREB basic region and leucine zipper can fold separately and undergoes a clear conformational change upon binding to different types of DNA elements (specific-half or full CRE and nonspecific sequences (Bentley et al., 2023). At the same time Q1-KID-Q2 affects the bZIP conformational landscape and subsequently modulates DNA binding, dimerization, and overall CREB transcriptional functionality (Bentley et al., 2023).

Nuclear localization signal (NLS) peptide−is a short peptide (RRKKK) located in between the basic region and leucine zipper domain of CREB protein (Figures 2, 3), and this NLS peptide facilitates the cytoplasmic CREB proteins to enter the nucleus. Therefore, contextual subcellular localization acts as a molecular switch on the transcriptional activity of CREB (Arnould et al., 2002; Hou et al., 2019). Under pathophysiological condition, epigenetic mechanisms and post-translational modifications, such as phosphorylation and SUMOylation, alter the subcellular distribution of CREB or even in mitochondrial matrix (Waeber and Habener, 1991; Acin-Perez et al., 2009; De Rasmo et al., 2009; Steven and Seliger, 2016; Lu et al., 2021).

CREB family transcription factors consist of multiple domains that play a critical role in regulating diverse cellular functions. Given that precise inter-domain interactions are generally required for the normal function of a multi-domain protein (Xia et al., 2023), the multiple domains of CREB are suggested to cooperatively control CREB-dependent gene expression via distinct domain-domain interactions.

## 3 Alternative splicing

The splicing of pre-mRNA is a fundamental process in eukaryotic gene expression. Alternative splicing, which contributes greatly to protein diversity, plays an instrumental role in regulating the activity of many transcription factors. Multi-exonic gene *CREB* undergoes extensive splicing in many animals like *Aplysia*, *Drosophila*, and human (Figure 2; Ruppert et al., 1992; Meyer et al., 1993; Yin et al., 1995b). CREB family genes produce multiple spliced isoforms that either enhance or repress gene expression, mediating modulatory function in different biological contexts (Bartsch et al., 1998; Sassone-Corsi, 1998; Blöcher et al., 2003, 2005; Poels et al., 2004; Sadamoto et al., 2010). In *Drosophila*, CREB proteins are identified as *dCREBA* and *dCREBB* (Yin et al., 1995a,b; Andrew et al., 1997; Rose et al., 1997). To date, *dCREBB* has been identified as 10 transcripts and 10 polypeptides (7 distinct); notable spliced isoforms include *dCREBB-a*, *-b*, *-c*, *-d*, *-e*, *-f*, and *-g* (Figure 3; Yin et al., 1994, 1995a; 1995b). Several studies confirmed that some isoforms showed remarkable functional differences. A gain-of-function study using CREBB-a and CREBB-b demonstrated that CREBB-a improves olfactory memory, while CREBB-b impairs it (Yin et al., 1994, 1995b). Accordingly, two distinct isoforms may function as a transcriptional activator or repressor (Yin et al., 1994, 1995b; Perazzona et al., 2004), while the specific function of other isoforms in a cellular context remains to be discovered.

The other CREB family protein, dCREBA is required for normal embryonic development (dorso-ventral patterning) and encodes only one type of protein isoform, which is less conserved to mammalian CREB, but shows high conservation to mammalian CREB3L1 (Smolik et al., 1992; Rose et al., 1997). However, recent studies on dCREBA confirm that it promotes protein synthesis-dependent long-term memory (LTM) formation (Lin et al., 2021). Apart from *Drosophila*, studies confirmed that in *Aplysia*, at least three different CREB isoforms function in a competitive manner in LTM formation through synaptic facilitation (Upadhya et al., 2004; Mohamed et al., 2005; Liu et al., 2008; Liu and Aguilera, 2009). In addition, *Lymnaea* CREB1 (Figure 2), a homologue of mammalian CREB, enhanced synaptic facilitation and produced activator and repressor isoforms whose levels of expression changed in a contextual/learning-dependent manner (Sadamoto et al., 2004, 2010, 2011). In a broader sense alternative splicing appears to increase the functional diversity of proteins from a single gene, which should be associated with the gain of new macromolecular interactomes as well as the evolutionary new traits (Wright et al., 2022). Therefore, alternatively spliced CREB gene variants, such as repressor or activator not only functionally exert different biological function, but also possess a fine-tuning regulation over transcriptional activity by facilitating or inhibiting the function of other isoforms (Lin et al., 2021).

## 4 Differential phosphorylation of CREB

CREB proteins contain a conserved cluster of kinase sites within the KID domain (Figures 1, 3) (Trinh et al., 2013; Thiel et al., 2021). A large number of studies have been conducted in the KID domain to define phosphorylation sites and to determine the physiological stimuli and protein kinases that induce phosphorylation. Also, one intriguing question is in what context, or how, phosphorylation modulates CREB transcriptional activity in a biological process (Gau et al., 2002; Kornhauser et al., 2002). So far, 14 phosphorylation sites observed in human KID include residues S89, S98, T100, S108, S111, S114, S117, T119, S121, S129, S133, S142, S143, and S156 (Figure 3 and Table 2; Sun et al., 1994; Shanware et al., 2007).

**TABLE 2:** Distinct phosphorylation of the KID domain and its effects on CREB function.

Phosphorylation sites		Human protein kinases	Effects on CREB function		References
**Human**	***Drosophila***		**Transcriptional activity**	**Underlying mechanisms**	
Thr-100	–	ATM, ATR	Decrease	Reduce the KID-KIX Interaction.	Shi et al., 2004
Ser-108	Ser-210	ATM, ATR, CK1, CK2	Decrease	Reduce the interaction of the CBP KIX domain with the CREB KID domain.	Saeki et al., 1999; Horiuchi et al., 2004; Shnitkind et al., 2018
Ser-111	Ser-213	ATM, ATR	Decrease	Reduce the KID-KIX Interaction.	Shi et al., 2004
		CK1, CK2	Decrease	Prevent DNA binding and reduce the interaction of the CREB KID with the CBP KIX domain.	Montminy et al., 1996; Shnitkind et al., 2018
		CK1, CK2	Increase	Higher activation potential in CRE-mediated transcription.	Saeki et al., 1999; Horiuchi et al., 2004
Ser-114	Ser-215	CK1, CK2	Decrease	Prevent DNA binding and reduce the interaction of the CREB KID with the CBP KIX domain.	Montminy et al., 1996; Shnitkind et al., 2018
		CK1, CK2	Increase	Higher activation potential in CRE-mediated transcription.	Saeki et al., 1999; Shnitkind et al., 2018
Ser-117	Ser-219	CK1, CK2	Decrease	Prevent DNA binding and reduce the interaction of the CREB KID with the CBP KIX domain.	Horiuchi et al., 2004; Shnitkind et al., 2018
Ser-121	-	ATM, ATR	Decrease	Phosphorylated CREB is unable to bind to CBP and consequently induce proteasomal degradation through ubiquitination pathway.	Shi et al., 2004; Dodson and Tibbetts, 2006
		PKC	Not determined	Increase dimerization. However, direct involvement of this PKC isoform as a CREB kinase is lacking.	Yamamoto et al., 1988
Ser-129	Thr-227	GSK-3α & 3β	Decrease	Decreased CREB binding to CRE motifs as well as reduced KID-KIX interaction.	Bullock and Habener, 1998; Grimes and Jope, 2001a; El Jamali et al., 2004; Hansen et al., 2004; Martin et al., 2005
		GSK-3α & 3β	Increase	Increased transcriptional activity of CREB in response to parathyroid hormone.	Fiol et al., 1994; Tyson et al., 2002
Ser-133	Ser-231	RSK1	Increase	Phosphorylation of CREB is induced by growth factor stimulus.	De Cesare et al., 1998; Xing et al., 1998
		RSK2	Increase	Phosphorylation of CREB is induced by growth factor stimulus.	De Cesare et al., 1998; Xing et al., 1998
		RSK3	Increase	Phosphorylation of CREB is induced by growth factor stimulus.	De Cesare et al., 1998; Xing et al., 1998
		MSK1	Increase	NGF-triggered phosphorylation of CREB in response to mitogens and cellular stress.	Deak et al., 1998; Wiggin et al., 2002
		MSK2 (RSK-B)	Increase	NGF-triggered phosphorylation of CREB in response to mitogens and cellular stress.	Deak et al., 1998; Wiggin et al., 2002
		DYRK1/ MNB	Increase	Phosphorylated CREB drives CRE-mediated gene expression during neuronal differentiation.	Yang et al., 2001; Tejedor and Hämmerle, 2011
		CaMKI	Increase	CREB regulates the integration of Ca^2+^ and cAMP signals.	Sheng et al., 1991; Sun et al., 1996
		CAMKII	Increase	CREB regulates the integration of Ca^2+^ and cAMP signals.	Dash et al., 1991; Sun et al., 1994
			Decrease	Failed to stimulate CREB-dependent gene transcription.	Sun et al., 1994
		CaMKIV	Increase	Activated CaMKIV, which is Ca^2+^ independent, directly enter the nucleus and induce CREB phosphorylation.	Dash et al., 1991; Sun et al., 1994
		PKA	Increase	Activation of PKA is absolutely dependent on Ca^2+^-stimulated adenylyl cyclase activity, and directly phosphorylates CREB without any cascade intermediates.	Gonzalez et al., 1989; Kandel, 2012
		PKB	Increase	PI3K-mediated activation of Akt/PKB increased CREB activity via a Ser-133 dependent mechanism.	Du and Montminy, 1998
		PKC	Unaffected	Increased formation of dimeric CREB-DNA complexes.	Yamamoto et al., 1988; Brindle et al., 1995
		BTK	Increase	BTK triggers the activation of the p38 MAP kinase and PKC isoforms and subsequently enhance phosphorylation.	Yang et al., 2004b
Ser-142	Ser-240	CaMKII	Increase	Phosphorylation of CREB mediates Ca^2+^-induced transcription of CREB target genes.	Dash et al., 1991; Sun et al., 1994; Wu and McMurray, 2001; Gau et al., 2002
		CaMKII	Decrease	Reduced dimerization and CBP binding and exert a negative effect on the transcriptional activity of CREB.	Dash et al., 1991; Sun et al., 1994; Wu and McMurray, 2001; Gau et al., 2002
Ser-143	–	CaMKII	Increase	Phosphorylation of CREB mediates Ca^2+^-induced transcription of CREB target genes.	Dash et al., 1991; Sun et al., 1994; Wu and McMurray, 2001; Gau et al., 2002

CREB is unavoidably phosphorylated in response to a broad spectrum of physiological stimuli that activate a variety of signaling cascades, including the Ras/ERK, PI3K/Akt, Ca^2+^, nitric oxide, and p38 MAPK signaling pathways (Figure 3; Xing et al., 1998; Arthur et al., 2004; Riccio et al., 2006). Many protein kinases found in diverse signaling pathways have been shown to modulate the CREB activity. These CREB upstream protein kinases include PKA, mitogen-activated protein kinases (MEKs), phosphoinositide 3 kinases (PI3K), protein kinase B (PKB)/AKT, protein kinase C (PKC), Ca^2+^/calmodulin-dependent protein kinase II (CaMKII), CaMKIV, RSK2, casein kinases I & II (CKI & CKII), ataxia-telangiectasia mutant kinase (ATM), HIK, mitogen and stress-activated protein kinase 1 (MSK1) and MSK2, SGK, TSSK5, and Dyrk1/MNB (Brindle et al., 1993; Enslen et al., 1995; Gubina et al., 2001; Wu and McMurray, 2001; Miyamoto, 2006; Yamashima, 2012). CaMKIV quickly phosphorylates CREB in a transient manner, while signaling from the MEK/ERK pathway induces a slower, but long-lasting phosphorylation of CREB (Impey and Goodman, 2001; Deisseroth and Tsien, 2002). RSK2 enhances robust CREB-dependent transcriptional response. In the case of MSKs, they mediate the late and prolonged phosphorylation of CREB (Xing et al., 1996; Seternes et al., 1999; Misra et al., 2002; Zhu et al., 2019), even though they are unable to enhance recruitment of CBP/p300. MSK-induced CREB phosphorylation downstream of MAPK signaling pathway is required for CREB-dependent gene expression (Naqvi et al., 2014). In addition, forskolin has been able to induce CREB-mediated transcription by consequential activation of adenylyl cyclase and PKA (Seternes et al., 1999; Misra and Pizzo, 2005).

Several reports also indicate that CREB phosphorylation can be promoted by multiple signaling molecules including N-methyl-D-aspartate (NMDA)-type glutamate receptors (Hardingham et al., 2001, 2002; Ghiani et al., 2007), cytosolic tyrosine kinase c-Src (Zhao et al., 2003), fibroblast growth factor receptor 1 (FGFR1) (Stachowiak et al., 2003; Hu et al., 2004; Fang et al., 2005), and estrogen receptors (Lazennec et al., 2001; Szego et al., 2006; Sharma et al., 2007). Major phosphorylation sites within the KID domain can be classified as follows:

Casein kinase site: In mammals and *Drosophila*, KID has multiple serine residues thought to be targets for casein kinases (CKs). For human CREB, CK sites 108, 111, 114, and 117 are homologous to residues 210, 213, 215, and 219, respectively, found in *Drosophila* dCREBB (Figure 3). However, human CREB possesses an extra CK site at residue 121. Studies have suggested CKs-mediated phosphorylation exerts functions in the nervous system (Blanquet, 2000; Horiuchi et al., 2004). Although it is reported that phosphorylation of the CK sites prevents DNA binding, while dephosphorylation allows favorable CREB-CRE binding, in that circumstance the exact consequence of phosphorylation for CREB activation remains to be determined (Lee et al., 1990; Armstrong et al., 1997; Pinna, 2002; Kim et al., 2016).

Glycogen synthase kinase 3 site: Ser-129 in human and Ser-227 in *Drosophila* are believed to be a substrate for glycogen synthase kinase 3 (GSK3) (Fiol et al., 1994). GSK3β-mediated phosphorylation of only Ser-129 leads to inactivation of CREB (Wang et al., 1994) through reducing the DNA binding activity (Figure 3; Bullock and Habener, 1998; Grimes and Jope, 2001a,b). However, in vitro experiments demonstrate that secondary phosphorylation by GSK-3 right after the phosphorylation of PKA plays a vital role for cAMP control of CREB (Fiol et al., 1994; Beurel et al., 2015).

Protein kinase A site: The mammalian Ser-133 residue is homologous to the *Drosophila* dCREBB Ser-231, which is critical for nuclear localization and transcriptional activity (Riabowol et al., 1988). A serine to alanine (S133A) mutation at this site does not affect binding to CRE sites of target genes, but results in impaired transcription (Figure 3; Du and Montminy, 1998; Shaywitz and Greenberg, 1999; Sakamoto and Frank, 2009). Typically, Ser-133 is phosphorylated by PKA to stabilize the alpha–helix domain of CREB, and allows for recruitment of the CREB co-activators CBP and p300 to induce transcription of target genes, either in homodimer or heterodimer form (Richards et al., 1996; Parker et al., 1998; Fimia et al., 1999; Jean-Rene et al., 2000; Naqvi et al., 2014). Similarly, Ser-133 residue is also phosphorylated by several CaMKs, even before being activated by calcium and calmodulin (Enslen et al., 1994; Matthews et al., 1994; Upadhya et al., 2004; Sakagami et al., 2005; Miyamoto, 2006; Yan et al., 2016).

CaMKIV site: Multiple studies have demonstrated that CaMKIV mediates the early phase of Ser-133 or dCREBB Ser-231 phosphorylation and enhances transcription. Interestingly, CaMKIV also phosphorylates the CREB co-activator CBP at Ser-301, which is considered to be a major target of CaMKIV phosphorylation, both *in vitro* and *in vivo* (Girardet et al., 1996).

CaMKII site: CaMKII can phosphorylate human CREB at Ser-133 (dCREBB Ser-231) and a second site, Ser-142 (dCREBB Ser-240) (Sun et al., 1994; Liu et al., 2013a). Phosphorylation of Ser-142 by CaMKII inhibits the dimerization of CREB and induces dissociation of CREB from the CRE (Parker et al., 1998; Wu and McMurray, 2001). Therefore, subsequent phosphorylation at Ser-133 and Ser-142 by CaMKII blocks the transactivation by inhibiting CBP protein interactions (Wu and McMurray, 2001; Deisseroth and Tsien, 2002). Interestingly, molecular manipulation of Ser-142 to alanine (S142A) increases CREB activity, which is induced by CaMKII- and CaMKIV-mediated phosphorylation, and also enhances intracellular Ca^2+^ accumulation, elevating CREB activity (Sun et al., 1994).

In contrast to other phosphorylation sites, Ser-133 is recurrently phosphorylated, followed by other serine, tyrosine, and threonine residues in the kinase inducible domain (KID), exerting a constructive influence on transcription (Quinn and Granner, 1990). However, there is considerable evidence that other types of CREB phosphorylation patterns can lead to the opposite effect on its transcriptional activity. CKs and ATM-mediated phosphorylation at serine residues 108, 111, 114, 117, and 121 inhibits CREB-mediated transcription by resisting co-activator recruitment and DNA binding, and even interfering with chromatin occupancy (Table 2; Shanware et al., 2007, 2009; Trinh et al., 2013; Kim et al., 2016).

Under rheostat conditions, membrane depolarization with high K^+^ level facilitates the influx of Ca^2+^, which induces CREB phosphorylation at Ser-133 and two additional sites, Ser-142 and Ser-143, promoting the activation of CREB (Kornhauser et al., 2002). Interestingly, phosphorylation of Ser-142 by CaMKII inhibits CaMKII-induced CREB activation, whereas the dual phosphorylation of CREB at Ser-142 and Ser-143 interferes with protein-protein interaction between CREB and CBP, raising the possibility that Ca^2+^ influx-mediated CREB activation in neurons may be independent of a CBP-based mechanism (Sun et al., 1994; Kreusser and Backs, 2014; Atsumi et al., 2024).

Apart from the regulation of physiological functionality, phosphorylation is also responsible for compartmental gating of CREB in different location in the cell. Even though no mitochondrial targeting sequence is present in CREB, Ser-133 phosphorylated form of CREB was found in inner mitochondrial compartment of adult rat brain (Fernandez-Silva et al., 1997; Cammarota et al., 1999). The phospho-CREB (Ser-133) forms a TOM (translocase of the outer membrane) complex with mtHSP70, and is transported into the mitochondrial matrix. Subsequently CREB binds to the CRE-like sequence in the non-coding region of the mitochondrial DNA (mtDNA) or D-loop of mtDNA (Lee et al., 2005; Marinov et al., 2014) and enhances the mitochondrial gene expression, such as ND2, ND5, and ND6 (Lee and Wei, 2005; Ryu et al., 2005). Interestingly any irregularities of CREB-mediated mitochondrial gene expression were shown to lead to neurodegenerative disorders (Lee et al., 2005).

In summary, it is well established that the phosphorylation of Ser-133 by PKA lead to nuclear localization of CREB and promotes the recruitment of co-activator CBP to initiate CREB dependent transcription. Other kinases, such as CaMKII, CaMKIV, RSK, GSK-3, MSK, and MAPK, phosphorylate Ser-133 and/or other neighboring serine residue(s), which affect overall transcriptional activity through the modulation of nuclear gating, DNA binding, dimerization, and recruitment of co-activators (Table 2). Taken together, differential and combinatorial phosphorylation of CREB appears to provide a versatile regulatory mechanism that plays essential roles in diverse biological processes (Deisseroth et al., 1996; Yang et al., 2004a; Restivo et al., 2009; Steven et al., 2020).

## 5 Homodimerization and heterodimerization

The basic and leucine zipper domains of mammalian CREB and dCREBB show a remarkably high similarity in their amino acid sequences. Approximately 78% of amino acids in leucine zippers and 92 % in putative DNA-binding domains of dCREBB and mammalian CREB, are identical (Hai et al., 1989; Pu and Struhl, 1991). CREB family proteins are organized into distinct domains that form active homodimer and heterodimer to assist interaction with the CREs, co-activators, and the basal transcriptional factors, thereby initiating robust transcription (Figure 1; Montminy, 1997). Functionally, the leucine zipper domain is responsible for dimer formation of CREB (Dwarki et al., 1990; Gui et al., 2012). Each mutation of three leucine residues L1, L2, and L3 in the leucine zipper domain shows significantly reduced DNA binding activity of CREB proteins, whereas the mutation of leucine residue L4 does not affect dimer formation and DNA binding (Loriaux et al., 1993, 1994).

Therefore, the dimer formation of CREB protein is more likely required for binding to CRE sites. In contrast, some mutations, which are introduced into the basic region, affect DNA binding activity drastically, but not dimerization of CREB (Dwarki et al., 1990). CRE binding proteins are structurally and functionally very similar, so they can dimerize with the activator protein 1 (AP-1) family members, such as the Fos, Jun, and ATF protein subfamilies (Hoeffler et al., 1988; Hai and Curran, 1991; Li and Green, 1996; Wu et al., 1998). For dimerization, CRE length plays significant roles. Studies have shown that the dimerization tendency of CREB family proteins is stronger at half CRE, due to higher competition with the CREB/ATF family at the complete CRE sequence. However, homodimers and heterodimers of ATF1 and CREM possess higher stability with full CRE binding than half CRE binding (Muchardt et al., 1990; Shaywitz and Greenberg, 1999).

Other bZIP family transcription factors, such as ATF2 or ATF3, can form heterodimers with c-Jun and c-Fos, but not with CREB or ATF1 (Hai and Curran, 1991; Vlahopoulos et al., 2008). Comparative study suggests that c-Jun/c-Fos and c-Jun/ATF2 & ATF3 heterodimers exhibit a higher affinity for full CRE sequences, but not at half CRE (Glick et al., 2016). It is mentionable that CREB protein fails to heterodimerize with Jun and Fos proteins (Benbrook and Jones, 1990; Jansen et al., 1997). Among the divergence in dimerization, the CREB/CREB homodimer exerts the longest half-life (10–20 minutes) with greater stability and potentiates the active transcriptional activity (Dworkin and Mantamadiotis, 2010). In addition to increasing or decreasing DNA binding affinity, different combinations of dimerization can also create diverse binding specificities, exerting their dual roles in activation and repression. For an example, human CREB5 protein, part of the CREB family, binds to CRE specifically with c-Jun or CRE-BP1 as a homodimer or heterodimer to function as a CRE-dependent trans-activator and act as an oncogene or biological marker in multiple cancers, particularly glioma (Wu et al., 2024). These clarify why different types of homodimers and heterodimers possess differential transcriptional and biological activity (Greschik et al., 1999; Vinson et al., 2006; Mulero et al., 2017).

## 6 Transcriptional co-activators

CREB-mediated activation of gene transcription depends on the recruitments of specific transcriptional co-activators, such as CREB-regulated transcriptional co-activators (CRTCs) (Iourgenko et al., 2003) and CREB-binding protein (CBP)/p300 (Altarejos and Montminy, 2011; Smith et al., 2021). The phosphorylation and dephosphorylation of CREB serve as a complex balance mechanism for co-activators recruitments and overall CREB transcriptional activity (Amelio et al., 2007; Ravnskjaer et al., 2013). Compared to wild-type CREB, mutant CREB[S133A] showed reduced capacity of CBP recruitment and compromised CREB activity in mice, even though it binds and occupies CRE sites (Gonzalez and Montminy, 1989). Surprisingly, mutant CREB[Y134F] act as a constitutive activator, which stimulate Ser133-dependent recruitment of CBP/p300 and overall transcription (Keyong et al., 2000).

Apart from CBP, CRTCs are activated through dephosphorylation and nuclear entry, subsequently form a transcriptional complex with CREB, CBP NONO, and KAT2B to promote the expression of CREB target genes (Conkright et al., 2003a; Ravnskjaer et al., 2007; He et al., 2009). Recruitment of CRTCs to the promotor enhances the association of CREB with the TFIID subunit TAFII130, rather than affecting the DNA binding activity of CREB (Conkright et al., 2003a).

The functional role of transcriptional co-activator CRTCs and CBP is tightly associated with cAMP and CREB activity (Wood et al., 2005, 2006; Talukdar and Chatterji, 2023). Phospho-KID of CREB interacts with the KIX domain of CBP, which is critical for the regulation of LTM and circadian activity (Chatterjee et al., 2020), whereas CBP was shown to improve cognitive impairments in an Alzheimer’s disease mouse model by increasing BDNF level and CREB activation (Wood et al., 2005; Nagahara et al., 2009; Creighton et al., 2022). CRTC1 regulates leptin-dependent glucose metabolism in diabetic state, CRTC2 endorses glucagon-induced gluconeogenic program in the liver, and CRTC3 controls lipid metabolism (Lv et al., 2016; Qiao et al., 2021). Moreover, these metabolic functions of CRTCs are actively correlated with CREB activity (Mair et al., 2011; Cui et al., 2021; Yoon et al., 2021).

## 7 Epigenetic, post-transcriptional, and post-translational control of CREB

The *CREB regulon* (set of CREB target genes) (Table 1) contains one or more consensus CRE sites in their *cis*-regulatory elements, which are involved in CREB-mediated regulation (Impey et al., 2004). A more extensive scan of the complete genome for CREB binding patterns revealed 1,349 mouse and 1,663 human CREB interacting sites in which the overexpression of 5,000 putative genes was reported upon CREB activation (Zhang et al., 2005). The list of presumed CREB target genes increases day-by-day, and includes genes that are involved in synaptic transmission, immune regulation, cell cycle & survival, metabolic pathways, and signal transduction (Zafra et al., 1992; Shieh et al., 1998; Tao et al., 1998; Mayr et al., 2001; Lonze et al., 2002).

In recent years, bioinformatics methods have been combined with microarrays and chromatin immunoprecipitation (ChIP)-based chromatin occupancy analysis (including ChIP-on-chip and SACO-serial analyses of chromatin occupancy), by which CRE and CREB-regulated gene expression was being studied across a variety of genomic regions (Fass et al., 2003; McClung and Nestler, 2003; Ghia et al., 2004; Zhang et al., 2005). However, the expression pattern analysis with a few CREB-dependent genes provide strong evidence to refute the idea that the expression of target genes is largely regulated by CRE and the phosphorylated CREB proteins (Zhang et al., 2005). Rather, CREB-mediated differential gene expression and functional regulation is controlled by diverse molecular mechanisms including epigenetic modifications, post-translational modifications, such as phosphorylation, methylation, SUMOylation, ubiquitination, glycosylation, and post-transcriptional modifications including microRNAs (miRNAs), and long non-coding RNA molecules (Lamarre-Vincent and Hsieh-Wilson, 2003; Pigazzi et al., 2009; Kaleem et al., 2011; Tan et al., 2012a; Liu et al., 2013b; Chen et al., 2014; Lin et al., 2014; Bordonaro and Lazarova, 2015; Deng et al., 2016; Noguchi et al., 2016; Wang et al., 2023).

Interestingly, CpG methylation of CRE promoters (TGACGTCA-containing CpG dinucleotides) both weakens the specific factor binding, and impairs transcriptional activity, eventually suppressing context-related gene expression (Iguchi-Ariga and Schaffner, 1989; Mancini et al., 1999; Iannello et al., 2000). In addition, HEK293T cells were examined for CREB binding affinity to CREs in both methylated and unmethylated states, and concluded that CREB binding within the genome depends on DNA methylation state in a tissue-specific way and the phosphorylation state of CREB (Jean-Rene et al., 2000; Keyong et al., 2000; Hiroshi et al., 2001) and also that binding to the CRE are not the primary regulators of target gene activation (Zhang et al., 2005). The transcriptional activity and targeted gene expression of CREB require the conscription of other transcriptional apparatus CBP/p300 (Arias et al., 1994; Kwok et al., 1994), CRTC (Iourgenko et al., 2003; He et al., 2009), and TAFII4 to the promoter site (Ferreri et al., 1994; Felinski and Quinn, 1999). CREB-binding protein CBP (CBP, also known as Nejire in *Drosophila*) and p300 both stand for histone intrinsic acetyltransferase (HAT) linked with transcriptional activators to the basal transcriptional apparatus (Gerritsen et al., 1997; Shankaranarayanan et al., 2001; Lu et al., 2002).

In particular, CBP acetylates the histone H3 at Lys-27, and non-histone protein CREB at three different lysine residues Lys-91, Lys-96, and Lys-136 (Paz et al., 2014; Shaukat et al., 2021). In combination, acetylation of histone H3 at lysine 27 alters the local chromatin environment and/or structure to promote DNA-binding, thereby enhancing CREB-CRE or CREB-CBP-mediated rapid gene expression (Bannister and Kouzarides, 1996; Barral et al., 2014; Li et al., 2015; Atsumi et al., 2024). Acetylation of three different lysine residues in CREB appears to enhance its interaction with both the CRE and CBP, promoting transcription of CREB-responsive genes, such as *BDNF*. Unexpectedly, mutations of three putative acetylation sites in CREB, which decreased interaction with the CRE or CBP, noticeably activated the gene expression of a cAMP-responsive element-dependent reporter (Lu et al., 2003; Bordonaro and Lazarova, 2015; Attar and Kurdistani, 2017; Guo et al., 2018; Akinsiku et al., 2021). As epigenetic regulation of chromatin accessibility modulates the CREB-dependent transcription, further study is needed to address the regulatory role of CREB acetylation (Kim and Kaang, 2017).

Ubiquitination and SUMO-modification can also affect the quality rheostat of CREB expression, target gene activation, and overall CREB function (Jeoung et al., 2022). Notably, hyperphosphorylated CREB can be subjected to ubiquitination and subsequent proteasomal degradation (Mu et al., 2011). SUMOylation in many cases inhibits the transcriptional activity by modifying subcellular compartmentalization and/or protein-protein interactions of transcriptional activators (Girdwood et al., 2004; Johnson, 2004). CREB contains three SUMO-modification motifs, such as EKSE (residues 154–157), RKRE (residues 284–287), and KKKE (residues 303–306) (Johnson, 2004; Du et al., 2008). Site-directed mutagenesis analysis showed that the K304R mutation in CREB diminishes the SUMO-modification and also prevents its nuclear localization (Comerford et al., 2003; Ryan et al., 2010). Intriguingly, CREB SUMOylation can be controlled by SUMOylation of AKT. Through the BDNF-TrkB signaling, AKT is phosphorylated at T380 and S473, and also SUMOylated at K276, enhancing its stability and kinase activity. Translocated active AKT directly phosphorylates SUMO1 at T76 and makes SUMO1 stable enough to SUMOylate nuclear proteins, such as CREB (Lin et al., 2016). Furthermore, there is a delicate crosstalk between phosphorylation and SUMOylation of CREB (Jeoung et al., 2022). Suppression of SUMOylation promotes the CREB phosphorylation, while preventing phosphorylation antagonizes the SUMOylation of CREB (Chen et al., 2014). Spatial memory training was shown to upregulate the expression of protein inhibitor of activated STAT1 (PIAS1) through phosphorylated CREB in the rat hippocampal CA1 region and then the SUMO E3 ligase PIAS1 then increased SUMOylation of CREB. As a summary, CREB phosphorylation seems to be an early event that sets off long-term memory formation, whereas later-induced CREB SUMOylation appears to be involved in maintaining long-term memory (Chen et al., 2014).

O-glycosylation of proteins is a potent, inducible post-translational alteration that exerts parallel effects, as like the phosphorylation’s event (Love and Hanover, 2005; Hart et al., 2007; Rexach et al., 2008, 2012). Due to the abundance of O-linked N-acetylglucosamine (GlcNAc) glycosylation observed in the brain, it is presumed to be a modulator of CREB activity and functionality (Khidekel et al., 2004; Vosseller et al., 2006). An *in vivo* study in the mammalian brain found the O-GlcNAc glycosylation of residues 256-261 (Ser-260 and Thr-256, -259, and -261) of the CREB Q2 domain, which impairs more than 50% of basal association with TAFII130/135 (Saluja et al., 1998). *In vitro* transcription studies showed that O-GlcNAc glycosylation significantly reduced the transcriptional activity of CREB, leading to downregulation of the basal expression of CREB target genes, such as *wnt2* and *c-Fos* (Rexach et al., 2008). These findings suggest that glycosylation acts like a constant repressor of CREB and deviates overall cellular function (Zachara and Hart, 2002; Khidekel et al., 2003; Zhang N. et al., 2022).

CREB has been reported as the direct target of several miRNAs, such as miR-1224, miR-128, miR-134, miR-144, miR-34b, miR-23a, miR-200b, and miR-301 (Tan et al., 2012a; Noguchi et al., 2016; Liu et al., 2020; Yang et al., 2021). In most of the case miRNAs negatively modulate the CREB expression and CREB-mediated signaling pathways through interaction with 3′-UTR of CREB (Steven and Seliger, 2016). Interestingly, CREB was also reported to regulate the expression of certain miR-9 and miR-373 that are involved in different type of cancer/tumor growth (Tan et al., 2012a,b; Zhang et al., 2013). The miR-466f-3p upregulation via CREB activation is associated with spatial learning (Wang I. F. et al., 2021, 2022).

In summary, it is obvious that the activation of CREB by phosphorylation and the presence of CREB response elements (CREs) are necessary, but not sufficient for the control of CREB-mediated target gene expression. A recent surprising report demonstrates that a dephospho-mimic mutant, S133A-CREB, can interact with CRTC family co-activators, leading to enhanced mesenchymal gene expression in mouse lens epithelial cells (LECs) (Zhang et al., 2023). This finding necessitates a re-evaluation of the role of phosphorylation-dependent CREB activation. However, in addition to phosphorylation-dependent regulation, epigenetic changes, non-coding small RNAs and post-translational modifications all cooperates with the transcriptional regulation of CREB to induce the selective expression of its target genes in different cell types.

## 8 Conclusion and standpoints

Given that the various cellular functions mediated by the CREB family transcription factors require CREB-induced expression of different combination of target genes, how differential gene expression by CREB is achieved is an intriguing question. Here, we present several regulatory modes of CREB family transcription factors that are associated with their pleiotropic functions in the nervous system as well as in various non-neuronal tissues. First, the extensively spliced CREB isoforms are classified as activators or repressors on the basis of functionality, correlating with the structural domain organization. Second, differential and combinatorial phosphorylation of CREB through the KID domain plays an essential role in modulating the transcriptional activity of CREB. A large number of protein kinases that were known to phosphorylate the KID domain function as major mediators of multiple signaling pathways activated in response to different types of physiological stimuli. This explains how several signaling pathways coupled with different cellular functions converge on and integrate with the CREB transcription factors. Third, homodimerization and heterodimerization of CREB proteins provide another regulatory mechanism underlying their differential transcriptional activity. Fourth, distinct transcriptional co-activators, such as CRTCs and CBP, are required for CREB-mediated gene expression. Fifth, epigenetic modifications, such as DNA methylation and histone acetylation, are also used to CREB- or CBP-dependent gene expression. Sixth, several miRNAs were shown to directly target and downregulate *CREB* genes and interestingly certain miRNA genes were reported to be regulated by CREB, suggesting a potential feedback loop. Lastly, in addition to phosphorylation, ubiquitination, SUMO-modification, and O-glycosylation contribute to the modulation of CREB expression, activity, and target gene expression.

Although great progress has been made in understanding the regulatory mechanisms of the CREB family transcription factors, there are still many unanswered questions. For example, what are the differences in the functions of each CREB isoform in neuronal and non-neuronal cells? Several CREB isoforms across species have been implicated in neuronal memory, but their specific roles as activators or repressors are unclear. Additionally, non-neuronal variants like human testicular htCREB and mitochondrial mitoCREB exist. However, the structural and functional differences between these isoforms remain poorly understood. Further research is needed to clarify these distinctions.

Differential and combinatorial phosphorylation of CREB through the KID domain plays an essential role in modulating the transcriptional activity of CREB. While cAMP and PKA were considered as the key modulator of CREB’s transcriptional activity, recent advancement confirms the involvement of another kinases and stimuli. It’s obvious that CREB phosphorylation appears much more intricate and precise regulatory mechanism than we expect.

Another interesting question is what effect the phosphorylation code has on other post-translational modifications, such as ubiquitination, SUMO-modification, and O-glycosylation. In general, differential gene expression in different types of cells can be regulated by modular and pleiotropic enhancers, distinct combinations of transcription factors, and epigenetic modifications. In this respect, additional researches are needed on other transcription factors and cofactors that work in cooperation with CREB, the CREB enhancer modularity, and epigenetic changes that are influenced by CREB-associated physiological stimuli. Since dysregulated CREB signaling are associated with a wide range of neuronal and non-neuronal diseases, the identification of CREB target genes directly responsible for the onset of these diseases will be of great help in developing their treatments.

## Author contributions

MC: Conceptualization, Data curation, Writing−review and editing, Formal analysis, Investigation, Resources, Visualization, Writing−original draft. MH: Data curation, Formal analysis, Investigation, Writing−review and editing. JL: Data curation, Writing−review and editing, Supervision, Validation. SJ: Data curation, Supervision, Validation, Writing−review and editing, Conceptualization, Funding acquisition.
